# The Association of Social Determinants of Health on Monitoring for Disease Progression Among Patients With Primary Open-Angle Glaucoma

**DOI:** 10.1167/tvst.14.3.15

**Published:** 2025-03-13

**Authors:** Kunal Kanwar, Rithambara Ramachandran, Joshua D. Stein, Chris A. Andrews, Azraa S. Chaudhury, Maryam Ige, Xueqing Zhou, Shikha Marwah, Yang Li, Charlesnika T. Evans, Abel N. Kho, Paul J. Bryar, Dustin D. French, on behalf of the Sight Outcomes Research Collaborative (SOURCE) Consortium

**Affiliations:** 1 Department of Ophthalmology, Northwestern University Feinberg School of Medicine, Chicago, IL, USA; 2 Department of Ophthalmology and Visual Sciences, University of Michigan Kellogg Eye Center, Ann Arbor, MI, USA; 3 Department of Preventive Medicine, Northwestern University Feinberg School of Medicine, Chicago, IL, USA; 4 Department of Veterans Affairs, Center of Innovation for Complex Chronic Healthcare, Hines IL, USA; 5 Department of Medical Social Science, Northwestern University Feinberg School of Medicine, Chicago, IL, USA

**Keywords:** primary open-angle glaucoma, social determinants of health, quality of care, optic nerve evaluation

## Abstract

**Purpose:**

To examine the association of race, ethnicity, and other social determinants of health (SDH) on receipt of optic nerve (ON) evaluation in accordance with National Quality Forum (NQF) and the American Academy of Ophthalmology (AAO) guideline-based metrics.

**Methods:**

This was a retrospective cohort study consisting of 13,582 patients with POAG receiving care across 12 tertiary care health. The odds of receiving ≥1 ON evaluations to monitor for glaucoma progression over 45 months of follow-up was evaluated.

**Results:**

White patients (61%) with primary open angle glaucoma (POAG) had more guideline recommended ON evaluations during the 45-month follow-up period, compared with Asian American (52%) and Black (53%) patients (*P* < 0.001 for both). More non-Hispanic patients with POAG (58%) had ON evaluations in all 3 time periods compared with persons of Latinx ethnicity (52%) (*P* = 0.045). The odds of undergoing ON evaluations were 17% lower for Black patients compared with White patients (odds ratio [OR] = 0.83; confidence interval [CI], 0.74–0.94), 56% lower for patients living in isolated rural communities (OR = 0.44; CI, 0.25–0.77) compared to urban areas, and 9% lower for patients residing in more impoverished communities (OR = 0.91; CI, 0.86–0.96).

**Conclusions:**

Racial and ethnic minorities and those residing in lesser affluent or rural communities are less likely to receive monitoring for POAG progression in accordance with NQF and AAO guidelines.

**Translational Relevance:**

This study aimed to determine the association between SDH and receiving POAG testing according to clinical practice guidelines, with a goal of identifying factors contributing to disparities in care. This should facilitate development of targeted clinical interventions for vulnerable patients with POAG while factoring technology, economic sustainability, and policy.

## Introduction

Primary open-angle glaucoma (POAG) is a leading cause of irreversible blindness, affecting more than 3 million person in the United States and 76 million individuals worldwide.^1^^–^^5^ Because of the often insidious nature of this disease, it can be challenging for patients with POAG to tell whether their disease is stable or getting worse without regular visits to eye care professionals who can perform ocular diagnostic testing (e.g., perimetry, optical coherence tomography, fundus photography) to check for structural or functional evidence of disease progression. Clinical practice guidelines put forth by the American academy of ophthalmology (AAO) and the National Quality Forum recommend that most patients should undergo at least two follow-up visits per year, including an annual evaluation of their optic nerve (ON) and retinal nerve fiber layer (rNFL) tissue to check for disease progression.^6^^,^^7^

Prior research has found that racial and ethnic minorities have an increased prevalence of glaucoma, first present for care with more advanced disease, and have an increased risk of experiencing blindness.^8^^,^^9^ A requisite step to reducing disparities and inequities in outcomes of POAG among racial and ethnic minorities requires enhanced understanding of factors that may limit vulnerable groups of minority patients from receiving care that is in accordance with established practice guidelines.^10^^–^^12^

This study is unique in its approach compared to previous studies, because we use an innovative approach that permits us to link detailed longitudinal clinical data on a large cohort of patients receiving eye care at academic medical centers throughout the United States with granular social determinants of health (SDH) data (e.g., patient income, level of educational attainment, urbanicity of residence, and the level of affluence of the community the patient resides in) to examine the potential association of the SDH on quality of care for White and Black patients with POAG. The medical centers in this study participate in the Sight Outcomes Research Collaborative (SOURCE) Ophthalmology Big Data Repository. This purpose of this study is to examine the social determinants of health and their association on receiving POAG testing in accordance with the clinical practice guidelines and quality metrics.

## Methods

### Data Source

This is a retrospective cohort study of adults with POAG from 12 tertiary-care health systems participating in the SOURCE Ophthalmology Big Data consortium (https://www.sourcecollaborative.org/) from across the United States. All SOURCE sites are on the EPIC (Verona, WI, USA) electronic health record (EHR) system and contribute data on all eye care recipients seen at their institution from the date they went live on EPIC to the present. The SOURCE repository includes patient demographics, diagnoses as determined by International Classification of Diseases (ICD-9 and ICD-10) billing codes, outpatient medications prescribed, and orders and charges for visits, diagnostic and therapeutic procedures as captured using Current Procedure Terminology (CPT-4) procedure codes. Although all the data in SOURCE is deidentified, patients are each assigned privacy-preserving tokens from Datavant, Inc. (San Francisco, CA) permitting longitudinal follow-up over time. SOURCE sites have contributed 10 years of longitudinal data spanning the timeframe of 2012–2022. This study was approved by the Northwestern University and University of Michigan Institutional Review Boards who determined this research is exempt from requiring patient consent, because the data has been de-identified.

### Inclusion and Exclusion Criteria

We identified all adults aged ≥ 18 years diagnosed with POAG in 1 or both eyes based on ICD-9 or ICD-10 billing codes (Supplementary Table S1). To permit us to assess glaucoma care quality, eligible patients were also required to have at least one visit to an eye care professional (Supplementary Table S1) during the 15 months in SOURCE after their initial POAG diagnosis, and a total of at least 45 months of follow-up with any health care professional in SOURCE after their initial POAG diagnosis. Having a total of at least 45 months of follow-up means that the patient must have had at least 45 months between their initial POAG diagnosis and their last contact date in SOURCE. Although the AAO preferred pratice pattern (PPP) recommends at least annual evaluations for patients with POAG, we considered a 15-month window instead to allow flexibility for scheduling conflicts that may have arisen when trying to adhere to an annual evaluation schedule. We excluded patients younger than 18 years old, glaucoma suspects or those with other types of glaucoma besides POAG, and patients with insufficient follow-up.

### Identifying Patients With POAG Receiving Monitoring of the Optic Nerve in Accordance With the Quality Based Guidelines

The National Quality Forum and the 2020 AAO PPP guidelines for POAG recommends that patients with glaucoma undergo evaluation of the optic nerve (ON) or retinal nerve fiber layer (rNFL) to assess for disease progression at least once every 12 months.^6^ We classified patients as fulfilling this requirement if there was evidence in SOURCE that the patient had at least one documented description of their ON, at least one documented cup-to-disc estimate, at least one charge for rNFL optical coherence tomography (OCT) (CPT 92133), or at least one record of optic disc photography (CPT 92250) in at least one eye during the time windows of interest. If there was an ON description present but it was simply designated as “normal,” at least one additional of the above-mentioned criteria were also required to provide sufficient evidence the patient received care in accordance with the clinical practice guidelines. This criterion was also used when monitoring patients during each of the 15-month intervals to assess whether each patient met the clinical guidelines to monitor for disease progression.

Because POAG is a chronic disease, we were not only interested in studying how well patients with POAG were being monitored in the first year after their initial diagnosis, but also two subsequent 15-month periods.

### Statistical Analysis

All analyses were performed using R, version 4.1.3. Continuous variables were summarized with means and standard deviations. Categorical variables were summarized with counts and percentages. We determined the proportion of patients demonstrating ≥1 ON evaluation in the first, second, and third 15-month windows. We computed the proportion with such evaluations in none of the three windows, only one of the three windows, two of the three windows, and all three windows. χ^2^ tests were carried out to see if pattern of ON evaluations in the 45-month follow up period differed by race, past research has demonstrated that race has been recognized to be associated with appointment nonadherence in multiple medical subspecialties, including ophthalmology.^13^^–^^15^

To better understand how other factors besides race were associated with receipt of an ON evaluations during the timeframes of interest, we created several multivariable logistic regression models. These included the following:

1) Model 1: a model to assess factors associated with the receipt of at least 1 ON evaluation in the first 15 months following a patient's initial POAG diagnosis in SOURCE.
2) Model 2: a model to assess factors associated with at least 1 ON evaluation during all 3 of the 15-month windows following the patient's initial POAG diagnosis in SOURCE, indicative of complete adherence to the practice guidelines.
3) Model 3: a model to assess factors associated with no record of any ON evaluation during all three 15-month intervals after the patient's initial POAG diagnosis in SOURCE, indicating total non-adherence to the practice guidelines.

The regression models generated odds ratios (ORs) with 95% confidence intervals (CIs).

For each of the models, we included the following predictors: age at initial POAG diagnosis, sex, race, ethnicity, type of primary health insurance, primary language (English, Spanish, or other), level of educational attainment (less than high school, high school diploma, bachelor's degree, or graduate degree), income by $10k (considered as a continuous variable), and number of dependents in the patient's household. Because the patient's level of educational attainment, income, and number of household dependents are not routinely documented in the EHR, we used privacy-preserving linkage software from Datavant INC. to link in these variables with the clinical data in SOURCE for each patient. These additional socioeconomic variables were provided by AnalyticsIQ (Atlanta, GA, USA). Collinearity was assessed, and all variance inflation factor values were close to 1 indicating not much multicollinearity among the covariates (see Table 6).

Additional predictors in the regression models included urbanicity of the patients residence based on rural-urban commuting area (RUCA) classification and the Distressed Communities Index (DCI). The RUCA codes classify U.S. census tracts into different levels of urbanicity using measures of population density, urbanization, and daily commuting times, and assign each zip code a value of 1 to 10 (1 being the most urban and 10 the most rural).^16^ We classified the RUCA codes into 4 categories (urban, large rural area, small rural area, and isolated small rural area).^17^^,^^18^ DCI is a metric developed by the Economic Innovation Group and is a validated estimator of a community's socioeconomic distress by 5 digit zip code.^16^ It incorporates seven metrics: unemployment, education level, poverty rate, median income, business establishments, job growth, and housing vacancy by combining measures from the U.S. Census Bureau Business Patterns dataset and the American Community Survey. Scores range from 0 (lowest distress score) to 100 (highest distress score).

## Results

### Sociodemographic Characteristics of the Study Sample

Our study sample included 13,582 eligible patients with POAG (Figure). The median number of eligible patients across the 12 sites was 851 (interquartile range [IQR] 277–1620). The mean (SD) age of the patients at their date of initial diagnosis of POAG in SOURCE was 67.7 (10.3) years, and included 7621 (56.1%) females, 8162 (60.1%) White patients, 4050 (29.8%) Black patients, 559 (4.1%) Asian Americans, and 452 (3.3%) persons of Latinx ethnicity. Approximately 60% of patients in this cohort had primary health insurance coverage from Medicare, and 23% had commercial health insurance. In addition, the proportion of White patients (25%) and Asian patients (35%) with commercial insurance was higher than the proportion of Black patients (18%) with the same insurance type. Black patients had a higher rate of “other” insurance than any other race at 22% (see Table 7). The mean (SD) income of the study sample was $91,700 ($83,500). The majority of the patients (80.8%) resided in urban communities and the mean (SD) DCI score of the group was 32.9 (27.9) (Table 1).

**Figure.**
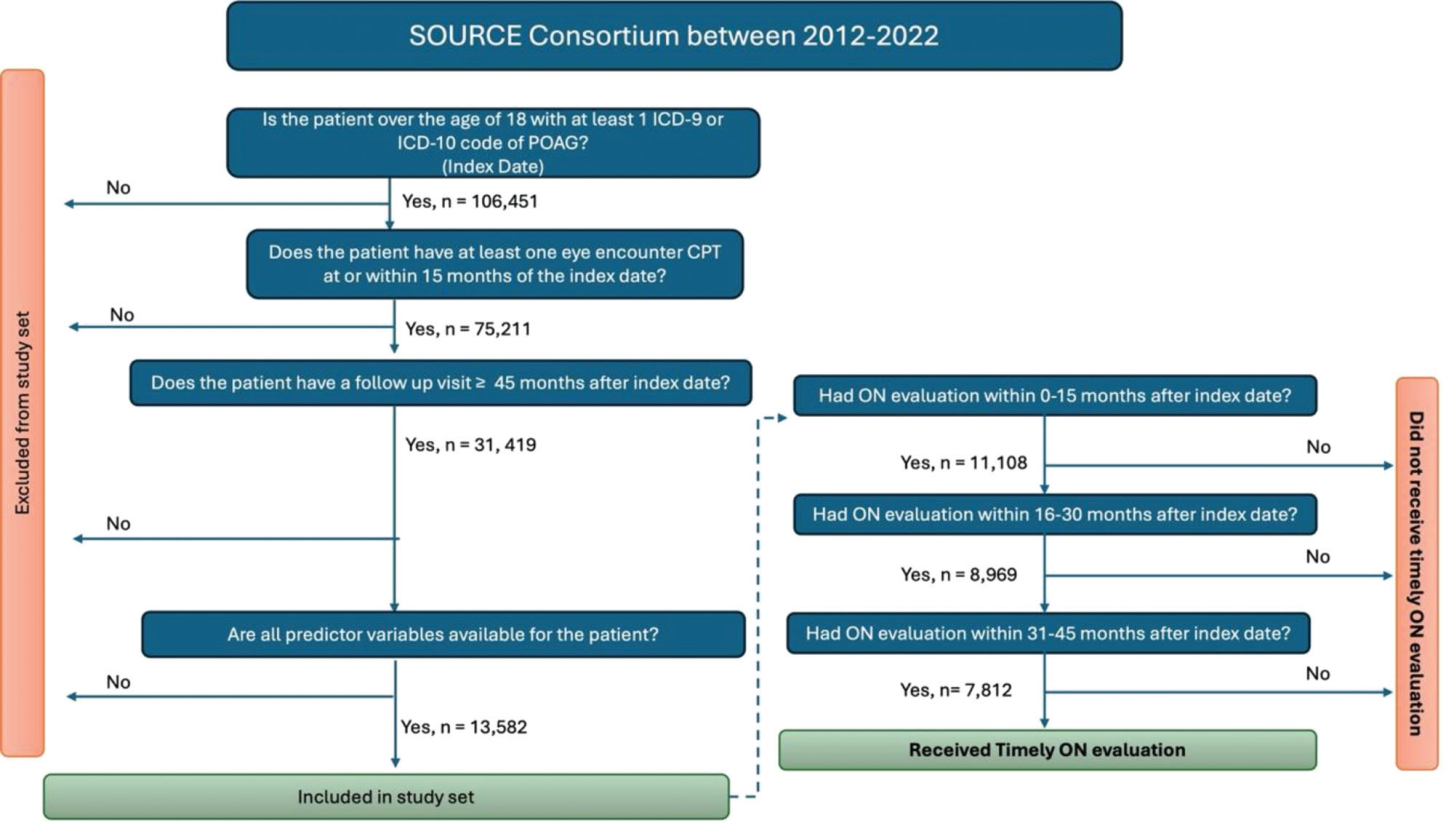
Selection diagram.

**Table 1.** Characteristics of the Eligible Patients With POAG


Total	13,582 (100%)			
Sex				
Female	7621 (56.1%)			
Male	5961 (43.9%)			
Race				
White	8162 (60.1%)			
Black	4050 (29.8%)			
Asian American	559 (4.1%)			
Other	811 (6.0%)			
Ethnicity				
Non-Hispanic	12,599 (92.8%)			
Latinx	452 (3.3%)			
Other	531 (3.9%)			
Primary Language				
English	13,326 (98.1%)			
Spanish	149 (1.1%)			
Other	107 (0.8%)			
Education				
Less than high school	997 (7.3%)			
High school diploma	6145 (45.2%)			
Bachelor's degree	4069 (30.0%)			
Graduate degree	2371 (17.5%)			
Health Insurance				
Commercial	3169 (23.4%)			
Medicaid	121 (0.9%)			
Medicare	8100 (59.6%)			
Other	2192 (16.1%)			
Urbanicity				
Urban	10,970 (80.8%)			
Large rural	182 (1.3%)			
Small rural	128 (1.0%)			
Isolated rural	82 (0.6%)			
No. of HH dependents				
0	13,225 (97.3%)			
1	210 (1.5%)			
2	70 (0.6%)			
3 or more	77 (0.6%)			

	**Mean**	**SD**	**Median**	**IQR**

Age at Initial POAG diagnosis (years)	67.7	10.3	69	62, 75
Income (by $10k)	91.7	83.5	71	41, 119
DCI Score	32.9	27.9	22	11, 54

HH, household.

DCI ranges from 0 (most affluent community of residence) to 100 (least affluent community of residence).

### Patterns of Receipt of Optic Nerve Evaluations for POAG

Among the 13,582 patients with POAG, 11,108 (81.8%) patients had ≥1 ON evaluation in the first 15-month period following the index date, 7812 (57.5%) had at least one ON evaluation during all three consecutive 15-month intervals, and 1532 (11.3%) patients had no record of any ON evaluations during the 45-month follow-up period (Table 2). When evaluating patterns of ON evaluations, the most common patterns across all races and ethnicities was consistent ON evaluation in all three time-intervals, followed by no ON evaluation in any of the time intervals studied (Tables 2 and 3). In other words, an individual was more likely to either receive optic nerve evaluations at all three time periods, as recommended by the guidelines, or not at all, rather than having inconsistent evaluations. The proportion of White patients (61%) with POAG who underwent ON evaluations during all three of the 15-month intervals was higher than Black (53%) and Asian American (52%) patients (*P* < 0.001 for both) and a greater proportion of non-Hispanic patients (58%) had ON evaluations during all three of the 15-month intervals compared to those of Latinx ethnicity (52%) (*P* = 0.05) (Table 3).

**Table 2.** Evidence of 1 or More ON Evaluations To Monitor POAG in Three Consecutive 15-Month Intervals After Initial Diagnosis

Months 0–15 From Initial POAG Diagnosis	Months 16–30 From Initial POAG Diagnosis	Months 31–45 From Initial POAG Diagnosis	
**X**	**X**	**X**	1532 (11.3%)
**X**	**X**	**✓**	265 (2.0%)
**X**	**✓**	**X**	221 (1.6%)
**X**	**✓**	**✓**	456 (3.4%)
**✓**	**X**	**X**	1307 (9.6%)
**✓**	**X**	**✓**	832 (6.1%)
**✓**	**✓**	**X**	1157 (8.5%)
**✓**	**✓**	**✓**	7812 (57.5%)

A check symbol indicates that the patient had 1 or more optic nerve evaluations during that particular 15 month time window. An “X” indicates that the patient did not have any evidence of an optic nerve evaluation during that particular time window.

**Table 3.** Receipt of 1 or More ON Evaluations to Monitor POAG in Each of the Three Consecutive 15-Month Windows After Initial Diagnosis, Stratified by Race and Ethnicity

	Race					Ethnicity			
Pattern	White	Black	Asian American	Other	*P* Value^*^	Non-Hispanic	Latinx	Other	*P* Value^†^
0/0/0	837 (10.3%)	449 (11.1%)	105 (18.8%)	141 (17.4%)	**<0.001**	1397 (11.1%)	73 (16.2%)	62 (11.7%)	**0.004**
0/0/1	141 (1.7%)	95 (2.4%)	19 (3.4%)	10 (1.2%)	**0.003**	249 (2.0%)	7 (1.6%)	9 (1.7%)	0.74
0/1/0	106 (1.3%)	94 (2.3%)	8 (1.4%)	13 (1.6%)	**<0.001**	208 (1.7%)	4 (0.9%)	9 (1.7%)	0.45
0/1/1	233 (2.9%)	177 (4.4%)	13 (2.3%)	33 (4.1%)	**<0.001**	424 (3.4%)	18 (4.0%)	14 (2.6%)	0.50
1/0/0	749 (9.2%)	435 (10.7%)	42 (7.5%)	81 (10.0%)	**0.02**	1227 (9.7%)	47 (10.4%)	33 (6.2%)	**0.02**
1/0/1	474 (5.8%)	282 (7.0%)	27 (4.8%)	49 (6.0%)	0.05	767 (6.1%)	28 (6.2%)	37 (7.0%)	0.71
1/1/0	636 (7.8%)	389 (9.6%)	55 (9.8%)	77 (9.5%)	**0.003**	1063 (8.4%)	40 (8.9%)	54 (10.2%)	0.36
1/1/1	4986 (61.1%)	2129 (52.6%)	290 (51.9%)	407 (50.2%)	**<0.001**	7264 (57.7%)	235 (52.0%)	313 (59.0%)	**0.05**

0, No receipt of an ON evaluation; 1, receipt of 1 or more ON evaluations.

* *P* value based on χ^2^ test comparing across all races.

† *P* value based on χ^2^ test comparing across all ethnicities.

A total of 7812 participants (57.5%) received at least one ON evaluation in each of the three 15-month windows, indicating complete concordance with NQF and AAO PPP guidelines. In unadjusted analyses, when comparing the patients who received ON evaluations in complete accordance with guidelines over the 45 months of follow-up and those who did not, race (*P* < 0.001), ethnicity (*P* = 0.045), urbanicity (*P* = 0.003), primary language (*P* < 0.001), educational attainment (*P* < 0.001), primary health insurance type (*P* < 0.001), income (*P* < 0.001), and DCI score (*P* < 0.0001) were all significantly different between the two groups. Factors that were not different between the groups were age at initial POAG diagnosis (*P* ≥ 0.9), sex (*P* = 0.6), and number of household dependents (*P* = 0.4) (Table 4).

**Table 4.** Characteristics of Patients Undergoing Annual ON Evaluations for POAG

	Not in Full Accordance With Guidelines During	In Full Accordance With Guidelines	*P* Value^*^
Total	5770	7812	
Sex			0.6
Females	3224 (55.9%)	4397 (56.3%)	
Males	2546 (44.1%)	3415 (43.7%)	
Race			**<0.001**
White	3176 (55%)	4986 (63.8%)	
Black	1921 (33.3%)	2129 (27.3%)	
Asian American	269 (4.7%)	290 (3.7%)	
Other	404 (7%)	407 (5.21%)	
Ethnicity			0.045
Non-Hispanic	5335 (92.5%)	7264 (93%)	
Latinx	217 (3.8%)	235 (3%)	
Other	218 (3.8%)	313 (4%)	
Primary Language			**<0.001**
English	5632 (97.6%)	7694 (98.5%)	
Spanish	80 (1.4%)	69 (0.9%)	
Other	58 (1%)	49 (0.6%)	
Education			**<0.001**
Less than high school	481 (8.3%)	516 (6.6%)	
High school diploma	2667 (46.2%)	3478 (44.5%)	
Bachelor's degree	1661 (28.8%)	2408 (30.8%)	
Graduate degree	961 (16.7%)	1410 (18.1%)	
Health Insurance			**<0.001**
Commercial	1117 (19.4%)	2052 (26.3%)	
Medicaid	67 (1.2%)	54 (0.7%)	
Medicare	3424 (59.3%)	4676 (59.9%)	
Other	1162 (20.1%)	1030 (13.2%)	
Urbanicity			**0.003**
Urban	4335 (96%)	6635 (96.9%)	
Large rural	75 (1.7%)	107 (1.6%)	
Small rural	60 (1.3%)	68 (1%)	
Isolated rural	47 (1%)	35 (0.5%)	
No. of HH dependents			**0.4**
0	5605 (97.1%)	7620 (97.5%)	
1	96 (1.7%)	114 (1.5%)	
2	35 (0.6%)	35 (0.5%)	
3 or more	34 (0.6%)	43 (0.6%)	
Age at Initial POAG diagnosis (years), mean (SD)	67.6 (10.9)	67.8 (9.9)	>0.9
Income (by $10k),^†^ mean (SD)	93.7 (94.5)	90.2 (74.4)	**<0.001**
DCI Score,^‡^ mean (SD)	35.3 (29.1)	31.3 (27.0)	**<0.001**

HH, household.

Receipt of optic nerve evaluation in accordance with clinical practice guidelines was defined as one or more records of documentation in the electronic health record of the optic nerve appearance, cup-to-disc estimate, retinal nerve fiber layer optical coherence tomography , or optic nerve photography in one or both eyes in the first, second, and third 15-month period after initial diagnosis of POAG.

* Pearson's χ^2^ test

† Defined by income predictor score in $10k.

‡ DCI ranges from 0 (most affluent community of residence) to 100 (least affluent community of residence)

### Factors Affecting the Odds of a Patient With POAG Receiving Optic Nerve Evaluations in Accordance With the Quality Metrics

#### Predictors of Receipt of at Least 1 Optic Nerve Evaluation During the 15-Month Follow-Up Period After Initial Glaucoma Diagnosis (Model 1)

The only predictor found to significantly increase the odds of having one or more ON evaluations in the first 15 months was Asian American race, which was associated with 43% greater odds of receiving an evaluation compared to White race (adjusted odds ratio [aOR] = 1.43; 95% confidence interval [CI], 1.07–1.92). Living in an isolated rural community, was associated with a 58% (aOR = 0.42; CI, 0.24–0.74) decreased odds of receiving one or more ON evaluations in the first 15 months after initial POAG diagnosis. Ethnicity, income, DCI, sex, number of household dependents, health insurance type, or educational attainment were not statistically significant (Supplementary Table S2).

#### Factors Associated With Undergoing Optic Nerve Evaluations During all Three 15 Month Intervals (Model 2)

Adjusting for other model covariates, compared to White patients, Black patients had a 15% lower odds (aOR = 0.85; CI, 0.75–0.95) of undergoing one or more ON evaluations in all three 15-month time periods. Residing in an isolated rural community was associated with a 51% reduced odds (aOR = 0.49; CI, 0.30–0.79) of receiving one or more ON evaluations in all three 15-month windows (Table 5^6^). For every 10 unit increase in DCI, the odds of a patient with POAG undergoing ON evaluations in complete accordance with established guidelines went down 3%. None of the other covariates in the model were statistically significant.

**Table 5.** Factors Associated With Receipt of at Least One ON Evaluation During all Three 15-Month Time Periods After Initial POAG Diagnosis

	Adjusted OR	95% CI
Sex		
Female	Reference	
Male	0.94	(0.86–1.02)
Race		
White	Reference	
Black	0.85	**(0.75–0.95)**
Asian American	1.23	(0.97–1.54)
Other	0.85	(0.69–1.05)
Ethnicity		
Non-Hispanic	Reference	
Latinx	1.03	(0.77–1.36)
Other	0.90	(0.73–1.11)
Primary language		
English	Reference	
Spanish	1.01	(0.64–1.62)
Other	0.67	(0.41–1.09)
Education		
Less than high school	0.85	(0.70–1.02)
High school degree	0.92	(0.83–1.02)
Bachelor's degree	Reference	
Graduate degree	0.97	(0.85–1.10)
Number of household dependents		
0	Reference	
1	1.13	(0.77–1.65)
2	0.78	(0.44–1.40)
3 or more	0.89	(0.51–1.53)
Health Insurance		
Commercial	Reference	
Medicaid	0.84	(0.56–1.28)
Medicare	1.05	(0.94–1.17)
Other	0.93	(0.79–1.09)
Urbanicity		
Urban	Reference	
Large rural	0.96	(0.69–1.33)
Small rural	0.90	(0.61–1.32)
Isolated rural	0.49	**(0.30–0.79)**
Age at initial POAG diagnosis	0.998	(0.993–1.003)
Income (by $10k)	0.995	(0.989–1.001)
DCI Score (by 10)	0.97	**(0.96,0.99)**

Bolded *P* values are statistically significant at *P* < 0.05. DCI ranges from 0 (most affluent community of residence) to 100 (least affluent community of residence)

**Table 6.** Variance Inflation Factor of Logistic Regression Model Evaluating Factors Associated With Receipt of at Least One ON Evaluation During all Three 15-Month Time Periods

	GVIF	Df	GVIFA^(l/(2*Df))
SITE_ID	17.039507	10	1.152319
Sex	1.035511	1	1.017600
Race	2.294647	3	1.148469
Age	1.500361	1	1.224892
Ethnicity	1.674413	2	1.137537
RUCA Urbanicity Score	1.089256	3	1.014351
Distressed Community Index Score	1.537005	1	1.239760
Primary Language	1.459391	2	1.099114
Income	1.380832	1	1.175088
Index Year	6.546763	10	1.098503
Education	1.231050	3	1.035252
Number of Children in the household	1.247135	3	1.037494
INSURANCE	2.127523	3	1.134085

#### Predictors of Complete Nonadherence to Optic Nerve Monitoring for POAG (Model 3)

Asian Americans had a 34% lower odds (aOR = 0.66; CI, 0.48–0.92) of having no record of any ON evaluations in the 45 months of follow-up compared to White patients. Furthermore, persons with POAG living in isolated rural communities had 167% (aOR = 2.67; CI, 1.38–5.15) greater odds of receiving no ON evaluations in the 45-month follow-up period after initial glaucoma diagnosis compared to those residing in urban communities (Supplementary Table S3).

## Discussion

We assessed the quality of glaucoma care as captured by routine monitoring of a patient's ON or rNFL tissue for a large cohort of patients with POAG across 12 tertiary-care U.S. health care systems and found that only 57% of the patients with POAG had been receiving ON evaluations in complete accordance with established clinical practice guidelines. Moreover, a sizable subset of the patients with POAG (11%) did not have a single record of an ON evaluation during the 45-month follow-up period since their index diagnosis visit. Black patients, those residing in lesser affluent communities, and those living in isolated rural communities were less likely to receive care in accordance with practice guidelines compared to White patients, those residing in more affluent communities, and those living in urban communities, respectively. 

Our findings are consistent with recent studies documenting racial/ethnic discrepancies in the utilization of ophthalmic services. Rasendran et al.^19^ examined data from the Medical Expenditure Panel Survey, a nationally-representative dataset of noninstitutionalized persons, and found that Latinx and Black patients had fewer outpatient visits than their non-Hispanic White counterparts. Halawa and colleagues^20^ examined Medicare claims data and found that compared to White beneficiaries, Black beneficiaries with glaucoma had lower frequencies of outpatient visits, perimetry testing, and rNFL OCT testing.^21^ Latinx beneficiaries similarly had lower rates of outpatient visits and ocular imaging compared to Whites. On the other hand, Asian Americans had higher rates of outpatient visits and perimetry.^20^ An examination of eye care utilization patterns in the AAO's IRIS Registry, similarly found that Black and Latinx patients with POAG were less likely to have eye exams and OCTs. Given that undergoing an assessment of the ON or rNFL OCT is nearly universally done during visits to eye care professionals, if racial and ethnic minorities are less likely to come in for eye care, this may explain why we are observing some differences in ON monitoring for POAG progression by race and ethnicity.

However, we hypothesize that factors affecting health care utilization are multifactorial and may be influenced by other SDH besides a patient's race or ethnicity. To the best of our knowledge, few studies have considered other SDH such as patient income, educational attainment, urbanicity, language, number of household dependents, and affluence of a patient's community of residence on the receipt of high-quality care for glaucoma. Many of these SDH are not routinely captured in the electronic health record as part of routine patient care activities to permit researchers to consider them.^10^ Leveraging sophisticated software that permits privacy-preserving linkages, we were able to link the rich clinical data in SOURCE to SDH data, permitting us to study the impact of selected SDH on glaucoma care quality, while accounting for other potentially important SDH factors in our models.

We identified that the community of residence where a patient with POAG resides is associated with their odds of receiving care that is in accordance with established clinical practice guidelines. For example, if there are two patients with glaucoma who are of the exact same sociodemographic profile and one of them resides in Flint, Michigan (zip code 48504, DCI score = 98), whereas another lives in an Troy, Michigan (zip code 48085, DCI = 7.3), which is an affluent suburb of Detroit, based on our modeling, the patient living in Flint has a 27% lower odds of undergoing monitoring of their ON in complete accordance with the guidelines. Similarly, a patient with POAG residing in North Lawndale, Illinois (zip code 60623, DCI 95.1), a socioeconomically depressed community in Chicago, is 23% less likely to undergo regular monitoring for glaucoma compared to a comparable patient living in downtown Chicago (zip code 60601, DCI 19.6). Ophthalmologists and optometrists may avoid practicing in depressed communities because of concerns about crime, lengthy travel from the healthcare provider's home, or other factors. Patients living in depressed communities may need to rely on public transportation to seek eye care services, and these services may be limited or unsafe, especially for persons who are visually impaired.

Another important finding from these analyses is that patients residing in isolated rural communities have a 56% reduced odds of receiving care that is in complete accordance with published guidelines for POAG and are at a 30% greater odds of never having received any ON evaluation in the 45-month follow-up period as compared to other patients living in urban communities. It has previously been identified that people residing in rural areas have lower rates of eye exams compared to their urban counterparts.^22^^–^^25^ Availability of eye care professionals may be limited in isolated rural communities. Often these communities also have limited public transportation so that patients with advanced glaucoma may need to drive lengthy distances, or require caregivers because they are unable to see to drive themselves. Given the reduced odds of a patient living in a rural area receiving guideline-based care, it is important to examine how limited access to public transportation may contribute to these disparities. In this specific study, because of the deidentification of our data, information regarding transportation was unavailable.

Health Insurance type is another SDH that should be considered when evaluating for the differences in ON monitoring between different groups. Previous literature has found that Medicaid recipients with POAG have lower glaucoma testing compared to those with commercial health insurance.^26^ Although this was not a finding in our specific study, it is important to note that insurance can affect the care a patient seeks and is able to access and thus may be a contributing factor to this inequity. Additionally, we found that there is a statistically significant association between health insurance type and race (Table 7). The lower rate of commercial insurance for Black patients may partially explain the lower adherence to ON evaluations observed among this ethnic group, because access to eyecare might be more limited among persons with other types of health insurance. In addition, Black patients had a disproportionately high rate of “other” insurance compared to other racial groups. This type of insurance might include insurance plans that may have limited access to glaucoma specialists, longer wait times, and financial barriers, all of which could affect adherence to glaucoma care guidelines.

**Table 7.** Association Between Insurance Type and Different Racial Groups

Characteristic	Commercial (n = 3169)	Medicaid (n = 121)	Medicare (n = 8100)	Other (n = 2192)
1: White	2042 (25%)	32 (0.4%)	5005 (61%)	1083 (13%)
2: Black	746 (18%)	61 (1.5%)	2358 (58%)	885 (22%)
3: Asian	194 (35%)	8 (1.4%)	258 (46%)	99 (18%)
Other	187 (23%)	20 (2.5%)	479 (59%)	125 (15%)

Our study was able to find the association of several SDH on receipt of ON evaluations in accordance with clinical practice guidelines, some of these may be modifiable. Specifically, an opportunity to improve glaucoma care quality for patients living in rural communities may be achievable with the expansion of teleophthalmology services including greater use of programs such as the Veterans Affairs TeleEye Care Services and greater use of technological advances such as home tonometry, home perimetry, and home OCT.^27^ Working with health care policymakers to incentivize eye care professionals to care for patients in underserved communities and companies to improve access to technological advances that permit home monitoring of patients living in isolated rural communities are ways to improve glaucoma care quality.^28^ In addition, targeted glaucoma screenings (TGS) may serve as another potential intervention for improving glaucoma care, particularly in communities with lower affluence. In previous studies, targeted glaucoma screenings were found to detect glaucoma/suspected glaucoma at a rate three times the national average, pointing to the effectiveness of these screenings to not only detect glaucoma but also target high-risk populations that may have otherwise not received healthcare intervention.^29^ TGS may be especially useful as it is well-documented that persons living in certain communities, even those with access to care, may have worse vision outcomes.^30^ Another community based approach for increasing utilization of eye care services is increasing patient education about POAG. The use of community outreach strategies to highlight the importance of regular eye care and to help individuals understand more about the progression of POAG may allow for improed care quality. It is also important to consider both culture and education while understanding the relevance of these results. Cultural influences, such as health literacy, health beliefs, and trust in the healthcare system, have been previously demonstrated to impact utilization of eye care services. Similarly, patients with lower educational attainment may struggle with health literacy. This can limit their understanding of disease management and the importance of regular evaluations. Although educational attainment was not a statistically significant SDH in our study, lower education has the potential to make navigating the healthcare system far more challenging even with efforts to follow up with care. We hope to consider cultural factors and education in future work if able to get access to data related to these factors.

### Limitations

Our study has several limitations. First, our analyses are restricted to tertiary care health systems in the SOURCE consortium. These findings may not be generalizable to patients receiving their eye care in private practice settings, Veterans Administration Medical Centers, or those residing outside of the United States. Additional analyses using other datasets are needed to assess the impact of SDH on glaucoma care quality in those patient populations. Second, some patients may be receiving a subset of their eye care outside of the health systems in SOURCE, and our analyses cannot account for this care. Although we have no reason to believe that this would vary by race, ethnicity, or other SDH, if it does vary by these factors, this may impact our findings. Third, our analyses did not consider disease severity. Patients with symptomatic vision loss from POAG may be more motivated for ON monitoring than those with asymptomatic or early stage of disease. Furthermore, some patients with very advanced glaucoma in both eyes have so much damage to their ONs and rNFL tissue that little may be gained from rigorous examinations of these tissues. Fourth, we required all patients to have received at least two visits to these health care systems to document presence of POAG and assess care quality. As such, all the patients in our analyses had the means and abilities to overcome at least basic barriers to care. Other patients with POAG who are more impoverished or vulnerable may be even less likely to receive care in accordance with established guidelines. Fifth, in our analysis we assess for whether patients have received at least one evaluation within each 15-month interval. However, this is the minimum recommendation by the AAO PPP guidelines. For patients with more severe glaucoma, a higher frequency of assessments may be needed to provide patients with appropriate care quality. Sixth, some EHR's allow for direct copying of prior ON exam findings to the current chart note, and as a result it is possible for some patients to have had visits where ON evaluation exam findings were documented but not truly received during that specific visit. This may affect the absolute rate of exams seen in the results; however, we do not believe that the rate of “copy forward” varied by SDH and thus we suspect that the relative rates of ON evaluation we observed are likely real.

## Conclusions

In this large multicenter study, we identified several key SDH besides race and ethnicity that are associated with glaucoma care quality including urbanicity of residence and affluence of the patient's community of residence. Policies incentivizing eye care professionals to go to evaluate underserved and lesser affluent communities, are needed.

## Supplementary Material

Supplement 1
