# Supplementary material for: The Association of Social Determinants of Health on Monitoring for Disease Progression Among Patients With Primary Open-Angle Glaucoma
Source: Transl Vis Sci Technol. 2025 Mar 13;14(3):15. doi: 10.1167/tvst.14.3.15 (PMC11918092; doi:10.1167/tvst.14.3.15)
Supplement: Supplement 1 [file tvst-14-3-15_s001.pdf]

Supplemental Table 1:

International Classification of Diseases ICD-9 and ICD-10 Billing Codes to Identify Patients with Primary Open-Angle Glaucoma Used for This Study.

ICD-9 codes
365.11
365.12
ICD_10 codes
H40.10X0
H40.10X1
H40.10X2
H40.10X3
H40.10X4
H40.1110
H40.1111
H40.1112
H40.1113
H40.1114
H40.1120
H40.1121
H40.1122
H40.1123
H40.151
H40.152
H40.153
H40.12

Current Procedural Terminology (CPT-4) Procedure Codes Identifying Visits to an Eye Care Professional

92002*	92004*	92012*	92014*	99201	99202
99203	99204	99205	99212	99213	99214
99215	99241	99242	99243	99244	99245
99304	99305	99306	99307	99308	99309
99310	99324	99325	99326	99327	99328
99334	99335	99336	99337		

*These are codes that are used exclusively by eye care professionals. All of the other CPT codes listed here were considered visits by eye care professionals only if the provider submitting the code was an ophthalmologist or optometrist.

Supplemental Table 2. Factors Associated with Receipt of At Least One Optic Nerve Evaluation During the first 15 Months Following Initial Primary Open-Angle Glaucoma Diagnosis

	**Adjusted Odds Ratio**	**95% Confidence Interval**
**Sex**		
Female	Reference	
Male	1.02	(0.91-1.15)
**Race**		
White	Reference	
Black	0.90	(0.77-1.06)
Asian American	1.43	**(1.07-1.92)**
Other	0.96	(0.72-1.26)
**Ethnicity**		
Non-Hispanic	Reference	
Latinx	1.14	(0.78-1.65)
Other	0.98	(0.74-1.31)
**Primary Language**		
English	Reference	
Spanish	1.34	(0.76-2.36)
Other	0.95	(0.53-1.72)
**Education**		
Less than High School	0.98	(0.77-1.26)
High School Degree	0.94	(0.82-1.07)
Bachelor's Degree	Reference	
Graduate Degree	0.97	(0.81-1.15)
**Number of Household Dependents**		
0	Reference	
1	0.81	(0.49-1.34)
2	0.68	(0.32-1.46)
3 or more	1.87	(0.64-5.46)
**Health Insurance**		
Commercial	Reference	
Medicaid	0.94	(0.55-1.58)
Medicare	0.90	(0.76-1.06)
Other	0.85	(0.69-1.06)
**Urbanicity**		
Urban	Reference	
Large Rural	1.09	(0.68-1.75)
Small Rural	1.10	(0.64-1.90)
Isolated Rural	0.42	**(0.24-0.74)**
**Age**	0.995	(0.989,1.002)
**Income (by $10k)**	1.003	(0.996-1.010)
**DCI Score (by 10)**	0.99	(0.97-1.02)

Bolded p-values are significant at P<0.05; DCI = Distressed Communities Index. DCI ranges from 0 (most affluent community of residence) to 100 (least affluent community of residence)

Supplemental Table 3. Factors Associated with No Receipt of Any Optic Nerve Evaluations to Monitor Primary Open-Angle Glaucoma During Three Consecutive 15 Month Time Periods Following Initial Diagnosis

	**Adjusted Odds Ratio**	**95% Confidence Interval**
**Sex**		
Female	Reference	
Male	1.02	(0.89-1.18)
**Race**		
White	Reference	
Black	0.85	(0.68-1.05)
Asian American	0.66	**(0.48-0.92)**
Other	0.94	(0.68-1.31)
**Ethnicity**		
Non-Hispanic	Reference	
Latinx	0.93	(0.61-1.43)
Other	0.98	(0.67-1.41)
**Primary Language**		
English	Reference	
Spanish	0.55	(0.29-1.07)
Other	0.56	(0.27-1.16)
**Education**		
Less than High School	1.09	(0.81-1.48)
High School Degree	0.96	(0.81-1.14)
Bachelor’s Degree	Reference	
Graduate Degree	0.87	(0.70-1.08)
**Number of Household Dependents**		
0	Reference	
1	1.06	(0.56-2.02)
2	0.93	(0.32-2.72)
3 or more	0.22	(0.03-1.77)
**Health Insurance**		
Commercial	Reference	
Medicaid	1.29	(0.70-2.36)
Medicare	1.03	(0.83-1.28)
Other	1.09	(0.83-1.43)
**Urbanicity**		
Urban	Reference	
Large Rural	1.06	(0.60-1.88)
Small Rural	0.71	(0.34-1.49)
Isolated Rural	2.67	**(1.38-5.15)**
**Age**	1.007	(0.999-1.016)
**Income (by $10k)**	0.99	(0.985-1.002)
**DCI Score (by 10)**	1.03	(1.00,1.07)

Bolded p-values are statistically significant at $P<0.05$; DCI = Distressed Communities Index. DCI ranges from 0 (most affluent community of residence) to 100 (least affluent community of residence)
